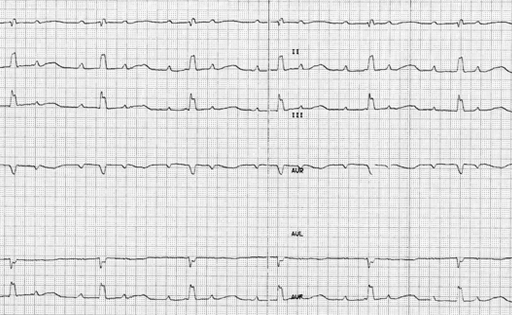# Second Degree Heart Block Associated with QT Prolongation

**Published:** 2010-02-01

**Authors:** Berna Saylan Cevik, Ayhan Cevik, Edide Tavli

**Affiliations:** Dr. Behcet Uz Children's Hospital, Pediatric Cardiology Department, Izmir

**Keywords:** Long  Q T syndrome, 2:1 AV block

## Abstract

Pseudo 2:1 AV block when sinus intervals are shorter than the ventricular refractory period has been reported with long QT syndrome (LQTS). We report the characteristics and treatment of a patient suffering from congenital LQTS with episodes of true 2:1 AV block. The pseudo 2:1 AV block relates to the extreme prolongation of ventricular refractoriness. Several histologic studies have documented abnormalities within the conduction system, including apoptosis. Because of the rare occurrence and poor prognosis of the LQTS with impaired AV conduction, international guidelines for diagnosis and treatment are needed.

## Introduction

Congenital long QT Syndrome (LQTS) with a prevalance of about 1 in 5000, is a familial disease with an autosomal dominant mode of transmission. It is a disorder of the electrical system of the heart due to dysfunction of the ion channels and involving the repolarisation process. In 12% of patients with LQTS, sudden death may be the first manifestation of disease and, importantly, in 4% it may happen in the first year of life. Syncope, ventricular tacycardia, or sudden cardiac death in the absence of structural heart disease is the typical presentation, beginning in childhood. The presence of a very long QT interval (> 00 msec), T wave alternans, 2:1 atrioventricular(AV) block, and inner ear deafness are proposed to indicate infants with a high cardiac risk. Fetal manifestations of LQTS may include sinus bradycardia, AV conduction block, and ventricular tachycardia [1]. The long QT syndrome (LQTS) is occasionally complicated by impaired AV conduction, mostly 2:1 AV block. Pseudo 2:1 AV block when sinus intervals are shorter than the ventricular refractory period has been reported with long QT syndrome (LQTS) [2]. This form of LQTS can manifest before birth or during neonatal life, and it is more sporadic than familial. It is usually an isolated disorder, although it can be accompanied by a variety of cardiovascular and other anomalies. In spite of different treatment modes, mortality is high  [3].

## Case Report

A seven years old girl was admitted with history of  three syncopal attacks. Her physical examination and laboratory findings were normal. There was no ion imbalance. Neurological examination was normal, her 12-derivation surface and 24 hour Holter ECG showed 2:1 AV block, QTc: 500 msec, nodal ectopic extrasystole (Figure1). Firstly we used beta blocker therapy (propranolol), but because of the serious cardiac side effects (hypotension, enhancement of AV block), we stopped the therapy, and a permanent pacemaker was implanted and she was discharged on propranolol therapy. She is now free of symptoms, 6 months after discharge.

## Discussion

Long QT syndrome (LQTS) is an inherited disorder characterized by a predisposition to the development of life-threatening ventricular tachyarrhythmias and prolongation of the QT interval on the electrocardiogram (ECG). The QT interval on the surface ECG is measured from the beginning of the QRS complex to the end of the T wave and represents the duration of activation and recovery of the ventricular myocardium. The corrected QT interval for heart rate (QTc) is considered to be less than 0.44 sec. Intervals longer than this, increase the risk of ventricular arrhythmias exponentially [4].

The extreme prolongation of ventricular action potential duration that occurs in some of the long QT syndromes may result in two forms of alternating activity of the heart: a "pseudo" 2:1 atrioventricular (AV) block and a T wave alternation, both of which are rate dependent. The pseudo 2:1 AV block relates to the extreme prolongation of ventricular refractoriness [5]. The block in this patient seemed unlikely to be related with a disease of conduction system, but is due to physiological block related to prolonged refractoriness.

Beta blockers are mainstay of therapy for prevention of cardiac events and implantable defibrillators for secondary prevention [3]. Patel et al. reported a such a similar case receiving an implantable cardioverter-defibrillator [6]. We used beta blocker theraphy (propranolol), but because of the serious cardiac side effects (hypotension, increased rate of AV block), we stopped the theraphy, and a permanent pacemaker was implanted and she was discharged on propranolol therapy.

Because of the rare occurrence and poor prognosis of the LQTS with impaired AV conduction, international guidelines for diagnosis and treatment are needed.

## Figures and Tables

**Figure 1:**